# Understanding the context for pet cat and dog feeding and exercising behaviour among pet owners in Ireland: a qualitative study

**DOI:** 10.1186/s13620-017-0107-8

**Published:** 2017-09-20

**Authors:** Martin J. Downes, Catherine Devitt, Marie T. Downes, Simon J. More

**Affiliations:** 1 Centre for Applied Health Economics, Menzies Health Institute Queensland, Nathan, QLD Australia; 2 Centre for Veterinary Epidemiology and Risk Analysis, School of Veterinary Medicine, University College Dublin, Dublin, Ireland; 3 School of Architecture, Planning and Environmental Policy, University College Dublin, Dublin, Ireland; 4 School of Medicine, Nathan Campus, Griffith University, 170 Kessels Road QLD, Griffith, 4111 Australia

**Keywords:** Cat, Dog, Behaviour, Attitude, Obesity, Exercise, Walk, Food, Feed, Diet

## Abstract

**Background:**

Pet cat and dog obesity contributes to increased risk of several diseases, including cancer and diabetes mellitus as well as a worsening of orthopaedic problems, and a reduction in survival rate. This study aims to develop a better understanding of cat and dog owners’ self-reported beliefs and factors that influence owner behaviour around feeding and exercising their pet cat or dog, as there is a lack of in-depth understanding in this area. Seven focus group discussions, with 43 pet owners in total, were conducted.

**Results:**

Pet owners often reported a perceived a low level of control over feeding; often undermined by other people feeding of their pet, their pets begging for food, and their pets attitude towards food. Treats were used in the absence of owner control over pet begging and emotional attachment, and to influence pet behaviour. The majority of participants had positive attitudes to pet exercise, which could be related to pet specific requirements, especially differences in cats and dogs. There were some negative experiences of stress associated with dog walking and fears over aggressive confrontations with other dogs.

**Conclusion:**

Feeding one’s pet is influenced by beliefs about pet specific needs, pet food and pet health, pet owners’ perceived control over feeding, and the implications for the pet owner. Pet exercise is influenced by beliefs about pet specific exercise needs, and the implications of exercising one’s pet for the pet owner. Understanding owner behaviours on feeding and exercise allows for a more targeted approach to preventing and treating pet obesity.

## Background

Obese and overweight pet dogs and cats are a growing problem in higher income countries, with studies showing that between 20 and 40% of pets are categorised as either overweight or obese in developed countries [1–6]. Obesity is the most common nutritional disorder in pet dogs and cats, leading to a series of adverse health consequences including an increased risk of various diseases, such as cancer and diabetes mellitus, a worsening of existing orthopaedic problems, and an overall reduction in survival rates [7–10].

### Explaining pet owner feeding and exercise behaviour

Obesity in pet dogs and cats has been associated with certain socio-demographic profiles, including older pet owners, female owners, low income households [2, 3, 5, 10], and there is increasing evidence to suggest a correlation between levels of obesity in humans and obesity levels in pets [11]. An unhealthy human-animal bond between owners and their pets and the over-humanization of pets can result in inappropriate feeding behaviour, which in turn influences pet obesity levels [2, 3, 10]. For example, pet owners often use treats as an expression of affection towards their pets, and unsurprisingly, this practice has been identified as a risk factor of pets being overweight [12–14]. Changes in feeding regimes and increased amounts of exercise have been an important component of weight management programs, especially in dogs [15, 16]. In addition, owner beliefs about their level of control over the pet’s behaviour, their knowledge about appropriate feeding, and their beliefs around pet exercise are important in explaining owner behaviour towards their pets. In other words, appropriate feeding and exercise are best explained by owner behavioural and control beliefs [17]. For example, owners of obese dogs were more likely to attach less importance to exercise and balanced nutrition than owners of normal weight dogs [17, 18]. Pet-specific perceptions are also important in explaining the reasons for pet obesity – the larger the dog is perceived to be, the greater the amount of food that the pet is given [18]. Perceived control over feeding and exercise is important. Kienzle, Bergler and Mandernach (1998) [18] showed that owners who believe they have control over feeding were more likely to have strong intentions to feed appropriately. Hence, owners who reported begging or stealing food from other pets, felt less in control of feeding their pet. Furthermore, a positive correlation exists between amount fed and owners’ perception of barriers to feeding appropriately [17].

As with perceived control over feeding one’s pet, owners that felt in control of their dog’s behaviour were more likely to exercise appropriately. The more that dog owners identified barriers to exercising their dog (e.g. lack of time, poor access to space to exercise one’s dog, etc.), the less likely was their intention to exercise [17]. These findings from the literature on pet ownership suggest that perceived control over pet attributes, behaviour and external factors, such as space, is an important indicator in explaining owner feeding and exercise behaviour. However, overall, it is the combination of pet owner beliefs and perceived control that underpins their behaviour, and influences pet health outcomes.

### Implications for veterinarians

There are implications for the role of the veterinarian in counselling and advising pet owners to follow appropriate behaviour for the health and wellbeing of their pet. Pet owners may not recognise that their pet is overweight, and this can result in discrepancies between the pet’s weight as perceived by the pet owner and that as evaluated by their veterinarian [19]. Differences also exist in expectations between the pet owners and the veterinarians regarding obesity management strategies [19]. These problems are accentuated by the reported differences in veterinary practice around weight evaluation with, for example, some dogs being infrequently weighed, bodyweight not commonly assessed and owners not always being told their dog’s weight classification [4, 20]. Veterinarians play an important role in providing information to pet owners on appropriate feeding and weight management for their pets; however, this may require additional methods of communication, rather than just providing verbal information [5, 21].

Understanding owner behaviours concerning pet feeding and exercise allows for a more targeted approach to preventing and treating pet obesity.

## Methods

The objective of this study was to identify the self-reported beliefs and factors that influence owner behaviour around feeding and exercising their pet.

### Study design

Research ethical approval was granted by the UCD Human Research Ethics Committee. Participants were required to sign a written form of consent. In this study, qualitative research methods, in the form of focus groups, were used.

### Participant recruitment

Pet owners were recruited through six different private veterinary practices (three Dublin city practices; one in county Wicklow, one in county Wexford (all located in south-east Ireland) and one in county Galway (west Ireland)). The practices selected were a convenience sample to ensure compliance and each agreed to participate in the study. The study was advertised through publication materials (posters and flyers) in the practices, prior to recruitment and participants volunteered to be enrolled in the study. Seven focus groups were conducted between April and June, with 43 participants in total; three to nine participants in each group. The first author (MJD) facilitated all seven focus groups.

### Data collection

Information on pet owner demographics (age, location, type of dwelling, and household composition) and pet demographics (type, neuter status and number of pets in participating households) were collected using a survey, prior to the commencement of focus groups.

A topic guide was used to direct focus group discussion. Questions were asked on views and decisions on pet neutering; feeding and weight control; and pet exercise. A topic guide was used during the focus group process as follows:

- Why do you have a pet?
- Why did you choose that type of pet?
- What are your views on neutering dogs and cats?
- What influenced your decision to have your pet neutered or not?
- What are your views on pet diets, both homemade and commercial?
- What factors influence the weight of your pet?
- How do you feel about exercising your pet?

Results on pet owners’ perceptions of pet neutering are reported elsewhere [22]. All focus groups were audio-recorded (with participant consent) and transcribed, and all identifiable information – such as names – were removed from the transcripts. The coding and the analysis process were assisted using Nvivo 8 (© QSR International Pty Ltd. 2007) qualitative data analysis software. During the initial analysis stages, the authors were cognisant of the existing literature that explains feeding and exercise behaviour, to assist in approaching the data deductively. The inductive-oriented guidelines and approach recommended by [23] Attride-Stirling (2001) for thematic analysis was subsequently employed. This involved a three stage process of first identifying basis codes, and using these codes to build a thematic framework comprising what are called organising and global themes. To help achieve inter-coder reliability, the authors discussed and agreed on the analysis process and resulting themes.

## Results

The socio-demographic profile and the number and type of pets in participant households is presented in Tables 1 and 2, respectively. Nine owners self-reported that their pets were overweight during the focus group discussion.

**Table 1:** Socio-demographic profile for participating pet owners (*N* = 43)

Socio-demographic variable	Frequency (%)	Ireland pet owners^a^ %
Age		
18–24	3 (7.0)	14.3
25–34	7 (16.3)	24.6
35–44	5 (11.6)	21.6
45–54	8 (18.6)	17.1
**55–64**	**14 (32.6)**	**14.15**
65+	6 (14.0)	8.25
Total	43 (100.0)	100
Gender		
Female	**30 (69.8)**	**50.7**
Male	13 (30.2)	49.3
Total	43 (100.00)	100
House type		
Apartment	1 (2.3)	1.4
Detached	**18 (41.9)**	**57.2**
Semi detached	13 (30.2)	28.6
Terraced house	9 (20.9)	10.0
Missing	2 (4.6)	2.8
Total	43 (100.00)	100
Household composition		
Lone parent with children	3 (7.0)	8.1
Married or Cohabiting couple	11 (25.6)	19.84
Married or Cohabiting couple with children	**13 (30.2)**	**59.3**
Mixed non-family household	8 (18.6)	4.13
One person	8 (18.6)	8.6
Total	43 (100.0)	100
Marital status		
Cohabitating	3 (7.0)	11.4
Divorced or Separated	2 (4.7)	8.4
Married	18 (41.9)	57.3
Single	**20 (46.5)**	**21.7**
Total	43 (100.0)	98.8^b^
Urban/Rural location		
Rural	15 (34.9)	38.1
Urban	**28 (65.1)**	**61.9**
Total	43 (100.0)	100

Bold = most frequent category

^a^Data taken from 10.1016/j.prevetmed.2009.07.005

^b^1.2% didn’t answer

This table is reproduced under Creative Commons licence © 2015 Downes et al. DOI: 10.7717/peerj.1196/table-1

**Table 2:** Profile of neutering (for cat, dogs, and both) among pet owners (*N* = 43)

Neuter status	Cat	Dog	Both cat and dog	Total
	*n* (%)	*n* (%)	*n* (%)	*N* (%)
Yes	8 (29.6)	9 (33.3)	10 (37)	27 (62.8)
Some	1 (12.5)	2 (25)	5 (18.5)	8 (18.6)
No	–	5 (62.5)	3 (37.5)	8 (18.6)
Total	9 (20.9)	16 (37.2)	18 (41.9)	43

This table is reproduced under Creative Commons licence © 2015 Downes et al. doi: 10.7717/peerj.1196/table-2

### Perceived control over pet feeding and pet behaviour

Suggesting a perceived low level of control over feeding, some owners reported that feeding was often undermined by other people’s feeding of their pet. Notably, this was particularly apparent among participants who declared they had overweight pets, compared with those who didn’t. Reported predominantly among dog owners, this lack of control takes place in households where there is more than one occupant and when the pet spends time with individuals other than the owner. Participants who had been brought up with pets and had pets for companionship especially identified these perceived control issues. These dog owners explained:

“My mother is a firm believer of spoiling dogs… I've been feeding them [food type] but my mother says that when the dog isn't eating the food, he doesn't like that food. She gives them all the bits from the dinner. I know when my mother has given them food because they won't eat … I'm fighting a losing battle with my mother”.

“The dog used to go up to my father’s; he was spoiled, grilled rashers. [My father would] say ‘oh yeah, [the dog] prefers boiled sausages this week, and chicken. [My father] used to give [the dog] everything, steak, and he was getting fatter”.

“It is very hard when somebody else is giving food [to the dogs], even after you tell them ‘no’, the vet said not to. My friend comes [to my house], I see her buttering bread, it’s for the dog… so now I have to put [the dog] out when we're eating… she keeps going under the table with food”.

This cat owner commented:

“[The cat] is 4 months. He is at the table every minute of the day. It is because my brother, my mother and my father are insisting on giving the bit of chicken or the bit of skin, ‘ah look he loves it’ ”.

As a means of addressing the problem; owners believed that having one person in the household with sole responsibility for feeding the pet was a certain way of maintaining a normal weight. For example, one dog owner mentioned the benefit of keeping a feeding calendar:

“I live with two other people, so we have a calendar… mark that she [the cat] has had her breakfast, she has had her dinner so that she is not getting fed by everyone”.

A perceived low level of control was also suggested by reported difficulties around monitoring food intake of individual animals if there were two or more pets in the household, and the potential for one pet eating the other pets’ food, and hence, the risk of pet obesity. A lack of control was also evident in participants’ statements on how pets (specifically, cats) *steal* food, or have a particular taste for some food items, without reoccurring reference to the owner’s control over what the pet was allowed eat.

“[the cats] like chicken and hamburger and whatever they can steal from you”

“If there’s leftover curry, [the cat] will eat that”.

“My cat eats an awful lot of tuna and just loves it. No matter what I give him, when the tuna comes out he would nearly take the door off the fridge just to get at tuna. He loves it every time”.

Pet-specific attitudinal beliefs are also apparent among participant statements that reflect perceived control. Cats are described as “grazers”, and are perceived as being more in control of their food intake, as opposed to dogs. In comparison, dogs are perceived as being less in control and more likely to overeat, therefore greater monitoring of dog food intake was reported as being necessary. Notably, a minority of owners who also declared their pet as being overweight, referred to concepts like animal genetics and animal nature to describe the weight of the pet, suggesting that pet-specific attributes influence feeding and obesity, rather than owner behaviour and owner control over feeding::

“It’s in [pet dog] nature, she is big boned and loose skinned and the other dog is the opposite. I think some dogs; it is in their nature and some cats. It is very difficult to control weight, you can feed them the exact same and one will put on weight and one will lose weight”.

Among participants who referred to their pet as being overweight, words other than obese or overweight were generally used, such as: “big boned”, “bit chubby” or “pudgy”. Direct mention of obese or obesity was mentioned on only five occasions during all of the focus group discussions.

Begging for food undermined owners’ perceived control over pet feeding. According to participating owners, a failure to ignore begging for food, and an emotional attachment to the pet, results in giving treats and food when the pet was present during pet owner meal times. This reflects a low perceived control over feeding, and is noted by most participants, but more in owners that declared their pet as being overweight than those that didn’t.

“[The dog] gets a little square of toast in the morning, when I'm having mine she sits beside me waiting for her bit to be given to her, that is her one regular treat that she gets…”.

“She's [the cat] is there looking at you, and you feel so sorry for her”.

“I always buy the chocolate drops especially for dogs because when you are watching TV on a Saturday, and you’ve got your chocolate out, … the dog is begging for theirs, have to have the treats for the dogs as well”.

Hand-feeding was also used where it is reported that the pet was underweight:

“One of my dogs loves to be hand fed, she is a very fussy eater, she’s very finicky. If she has not eaten for two days, I just have to hand feed her…”.

Owners reported how pet treats were often used as a way of influencing pet behaviour, for example, tempting the pet into the owner’s house, or encouraging greater food intake. Arguably, the use of treats may compensate for a low perceived control over pet behaviour:

“[The cat] won't come in [into the house], so the only way I can get him in is by tuna, I only give him a little bit, maybe a tablespoonful, he gets that quite often, tinned tuna, normal that I would eat… I need to get him in because he goes out the front [of the house] and his days will be numbered”.

“To get her [the cat] to be tempted to want to eat her dinner, you have to add a desert spoonful of the wet food as well”.

Similarly, treats were also used to reward particular types of pet behaviour, behaviour that is perceived as appropriate by the owner:

“When I'm leaving [the dog] behind, its standard practice, she has to get a dog biscuit to mind the house. She hasn't turned the house up yet, so it must be working”.

“I wanted to have something [in the diet] just for the sake of having something different. [The cat] is on heart tablets at the moment, so he gets the tuna. And he gets that with tablets, as a little treat. A reward for taking his tablets”.

However, owners of working (breeding, farm, and security) dogs believed one of their main motivations for feeding was to keep their pets healthy.

“... they're doing ok and are in good condition.”

### Beliefs about pet food and associated pet health outcomes

The types of food used was influenced by pet owner beliefs of what is necessary for good pet health and wellbeing, and this was influenced by how owners perceive the tangible health of their pet, and what was required to maintain this health:

“I think the cat is allergic to a lot more… we put her on dried food....but if she doesn't like it, she won't eat it. So I tried putting her on some other kind of wet food. It's for her stomach, there's no meat, there's no milk & all that type of thing. She's very picky”.

This owner explains that meat was given to lactating and pregnant female dogs, “but otherwise [the dogs] get just the nuts, they're all in good conditions, with shiny coats, if there was any problems I would change the diet, I have no skin trouble”. Again noting how healthy her cat was, this owner explains that the cat “lives on [food type], he gets it dry and some canned and he is very happy with a little bit of fresh chicken, his coat is very healthy, he’s fine”. Discussion was had on the effectiveness of current, popular diets in comparison to past diets (comprising of household leftovers, and vegetable waste):

“[the dog] lived to a great old age and he got nothing only what came out of the house, big pots of spuds [potatoes] put on for them”.

“I had another [type of dog]. He only got scraps and he lived to be 16 years or maybe 17”.

Comments reflect a possible distrust of processed pet foods, especially as past diets prepared at home by the owner did not seem to impact negatively on the pets’ health. These statements show that positive perceptions of pet health, based on an evaluation of tangible signs, reinforce pet owners’ positive beliefs around feeding behaviour.

The importance of convenience for the owner featured in a small number of comments on choice of diet for their pets. Comments related to; convenience around food preparation, environmental (housing) requirements, longevity of particular food types, and cost. The following were examples of quotes reflecting the influence on convenience on owner’s choice of diet:

“It’s cheaper to get mince-meat and cook it up. In [retail outlet], you get the cheap reduced sections of chicken. It’s probably better than the dog food. Sometimes, I go to [retail outlet], and get the tins of dog food for them. It works out cheaper, feeding them the mince than the [food from the] tins…”

“The advertisement on the bag, with the indoor formula assuring that low stool odour - when you live in a small flat in [other EU country], it is important…”

“It is convenient. The reason that I started on the [food type] is that you could get it in 10 [kg] packets which, with only two cats to start off, it is a lot of cat food. It would last you a while, don’t have to buy cat food every day. You can have it delivered. It turns up a week later with all your cat food. They might think you are odd buying 40 or 50 kg of cat food at a time but the bags don’t go off for a year or so, throw them under the stairs”.

### Beliefs about pet specific exercise requirements

There were clear differences between owners’ beliefs towards the exercise needs of cats, and those of dogs. The general consensus among pet owners was that cats tend to exercise themselves and exercise can be facilitated through play. Cats were generally regarded as sedentary animals with occasional bursts of skittish activity of running and playing, and this was considered by most as sufficient. A minority of cat owners did play with their cats, or reference was made to other individuals in the household playing with the cat. Exercise was regarded mainly for owner enjoyment and to facilitate social interaction with the cat, rather than as a means to facilitating exercise.

“Cats get their own exercise because they chase one another. Tearing up and down the stairs, in the bathroom, and wine glasses being broken… they get their own exercise themselves”.

“We take mice out and balls in the kitchen, so the cat does play with things himself in his own time, and he'll run up and down the garden, other times he'll just walk. I don't really have a routine of exercise; it's up to him [the cat]”.

Only one cat owner referred to a desire to exercise her cat; however, she explained: “I’m afraid what people would think”. A greater number of dog owners agreed that they needed to be actively involved in the exercise of their dogs. The majority of dog owners reported exercising their dog at least once a day, most commonly by walking or swimming. For some, reference to specific dog breeds reflects owners’ beliefs of what the dog requires to stay healthy, with emphasis placed on the importance of space.

“[The dog] tends to be a little bit chubby, but I think the [dog type] do tend to be chubby. I’m just trying to keep it down. She gets a lot of exercise, plenty of swimming and running on the beach every day”.

However, as with cats, other participants reported that active play is sufficient exercise, particularly when the dog has access to space and play activities.

“No, I don't need to [exercise the dog], the [type of dog] plays football, she exercises herself, they just run around, any of them will go hunting, after birds rats, anything, rabbits whatever, and they'll run around the field”.

In general, exercise regimes of dogs were influenced by owners’ beliefs of what their pet requires for development, wellbeing and health. Similarly, pets with particular health needs, and/or older pets were often excused from engaging in exercise.

“The dogs at home are not lazy, the only one who is; a bitch of 14 years and she's deaf so I don't mind her sleeping a lot, but the others are very active”.

For others, exercising the dog was perceived as necessary in controlling the dog’s energy and perceived contentment levels.

“[The dog] has to have [exercise] himself, he'd go mental otherwise”.

“[The dog is] a bundle of energy… like all dogs once they're walked, they're much calmer for the rest of the day”.

Allowing the dog to roam without being attached to a lead, and allowing dogs to play with other dogs, was regarded as being sufficient for exercising high energy dogs. Dog owner comments reflect what was regarded as important for exercise.

### Perceived control over pet exercise

Overall, the majority of participant comments reflected positive attitudes to pet exercise, translating into a high level of perceived control in exercising their pet. Interestingly, this was noted both by participants who declared they had overweight pets and those that didn’t, with some comments particularly from cat owners, reflecting owners’ perceptions of exercise appropriateness for their pets.

“they're [the cats] not ‘overweight’ overweight, but they are slightly pudgy cats... I feed them two and half times a day, and that seems to be fine. They both have that round cat shape when they are sitting but, I suppose with a pet because they're not having to hunt or anything like that they're naturally going to be a bit more prone to being carrying weight cause they're not hunting for their food anymore. And a cat because it’s smaller, if you have stairs in the house that is pretty much their exercise they’re running up and down the stairs… they're only little there not like a dog”

“I find with the cats they get their own exercise because they chase one another. You know they are tearing up and down the stairs, in the bathroom and wine glasses being broken…. they get their own exercise themselves”

For dog owners, exercise for their dogs is associated with daily routine, in some cases, the owners desire for exercise, and how exercise is viewed as calming the dog.

“the rest [of the dog] get fed once a day, the weight is great because they walk, my husband could go out for hours with them, he takes 2 or 3 [dogs] out at a time”

“I think overall because she [the dog] likes to be outside so much she gets sufficient exercise.”

“One of the reasons I got her [the dog] was to make me exercise, I knew that she would need the exercise so if she's getting it I would get it too. And that’s worked... I do try to see that she gets a good hours exercise every day and if not two shorter sessions I try to see that she gets plenty of exercises, if she meets other doggy pals, that’s the best, cause she runs with them and gets ten times as much exercise as her walk”

“A couple of miles and then at night they’d [dogs] get another 4 miles, and get a bit of exercise myself”

“I have a West Highland Terrier and she insists she’s going for a walk, when I'm working I walk her in the evening, and when I'm off it has to be after breakfast, she'd be happy even if I walked her in my dressing gown, she's a bundle of energy and that’s of course like all dogs once they're walked, they're much calmer for the rest of the day”

“I think exercising is the biggest key to keeping the weight down, I think you could feed your dog till the cows go home, as long as you bring them out for their regular exercise it definitely makes a big influence on their weight you know”

“… the routine is twice a day and he has to have it himself, he'd go mental otherwise, he knows, I think since we've always done it since he was a pup he knows the times and is ready to go when ever your ready to go. He's good like that, he enjoys it”

However, although only reported by a minority of participants, exercise routines were undermined by dog owner experiences of stress. This was associated with fears over aggressive confrontations between dogs and dealing with behaviours that were difficult to manage (such as pulling on the lead).

“I find that stressful! ... Another dog coming or whatever, I just think - Oh this is murder! I wouldn’t enjoy myself as well…”

“The dog is strong on the lead and she has never really copped on to that, we never properly trained her so she does pull a bit, especially if she sees other dogs so it's not the most comfortable thing, and she doesn't get that much exercise from a walk”.

“It’s stressful because the little terrier, ever since she was a pup, bites my legs, she gets really excited and she only does it to me. When she gets outside the door, she goes into play stance and then bites, and even the Rottweiler, he’s kind of looking at her and hitting her, tying to protect me from being bitten I suppose. Dragging me, well she is so small she doesn’t drag but she will try to pull me down the road”.

## Discussion

### Overview of results

This study used qualitative research methods to explore the self-reported beliefs and behaviours of pet owners in Ireland, around feeding and exercising their pets (cats and dogs). The authors have found no other study in Ireland that has looked at feeding and exercise behaviour among pet owners. Overall, the results suggest that attitudinal beliefs and perceived levels of control over the feeding regime, control relating to pet behaviour, and the perceived ease of feeding and exercise, are useful in explaining pet owners’ feeding and exercise behaviour. Similar to the findings of Rohlf et al. (2010) [17], subjective norms (i.e. social pressures and the level of motivation to follow these) are not as apparent in pet owner explanations on feeding and exercising of their pets, and instead, behavioural beliefs and perceived control are important in explaining feeding and exercise practices. Exercise behaviour draws on beliefs about what the specific pet (i.e. cat or dog) requires. Cats were regarded as being self-sufficient, whereas exercise regimes for dogs were influenced by owners’ beliefs of what their pet dog requires for development, wellbeing and health. Overall, participant comments reflect positive attitudes to pet exercise and only a minority of pet owners referred to difficulties experienced while exercising their dog. Interestingly, barriers such as cost of pet food and the negative implications of exercising one’s pet are only reported by a minority of pet owners.

Understanding pet feeding requires a more nuanced, complex perspective. The feeding of one’s pet draws on specific beliefs and perceived levels of control regarding pet-specific behaviours, what is needed for pet health and wellbeing, and how pet owners perceive tangible health signs. The reported ease of maintaining a normal weight in pets is made difficult by a perceived low level of control over the feeding behaviour of other individuals who have access to the pet. This is evident in participants’ statements on how pets (specifically, cats) “steal” food, or have a particular taste for some food items, without much reference to the owners control over what the pet is allowed to eat. A low level of control associated with begging, stealing food, and pet behaviour; treats are used to influence and reinforce certain behaviours. With regards to feeding and exercise, there were very few differences between those who declared they had over weight pets and those that had normal weight. The key difference being that, owners with overweight pets placed greater emphasis on the role of genetics as a cause for their pet’s size and weight.

Owners with overweight pets also used treat giving as a means of expressing emotional attachment to their pets. These results are in line with those observed by Rohlf et al. (2010) [17], regarding the impact of low level of control on owner feeding behaviour, and the potential for pet obesity, as well as the relationship between ambivalent beliefs about feeding, feeding behaviour and the risk of pet obesity. Reasons for owning a pet and the relationship with their pet may also be a factor in determining attitudes towards feeding pets. Participants with working dogs felt that they fed their pets to keep them happy; whereas, participants who had been brought up with pets and had pets for companionship identified controlling diet was an issue. This is in line with other findings, where owners considered the word treat to be food rather than any other form of pleasure for a pet [24] and the relationship of the human animal bound can contribute to the development of problem behaviours and overfeeding [25].

Given the predominance of these two areas in explaining pet owner feeding and exercise behaviour, the following discusses the implications for veterinary advice on responsible pet ownership, and weight control.

### Implications for veterinary advice and weight control initiatives

Veterinarians are in a unique position to communicate with animal owners about animal health [25]. In order to address obesity, it is important for veterinarians to understand why pet owners behave the way they do when feeding and exercising their pets. This understanding will assist veterinarians in tailoring communication strategies and initiatives around weight control [21]. Results show that beliefs about pet specific characteristics, pet needs, and the resulting perceptions of pet health and welfare are important in explaining why particular diets are used. Factors such as palatability and diet performance affect owners’ perception of food [26]. In this study, pet health outcomes are important in reinforcing positive beliefs about certain feeding regimes and food types. For example, healthy skin and hair condition are tangible outcomes that justify choosing particular diets for owners. Understanding owner beliefs is important for weight control initiatives and in order for owners to respond appropriately they need to believe and acknowledge that the pet is overweight [17]. This involves recognition of the obesity problem, followed by preparing for change in feeding and exercise behaviour and subsequently, implementation and maintenance of a disciplined programme of weight reduction [14]. Owner education and motivation is also crucial [16].

The results show differences in beliefs towards the exercise needs of specific pets, with dogs receiving some exercise, and play being deemed sufficient for cats. While owners do not necessarily see the need to exercise their cat, time spent playing with a cat has been shown to be an effective method of assisting weight control [12]. This is a feature of pet cat owner interaction that can be further developed by veterinarians as part of a weight management program; by explaining the benefits of playing with a pet cat, both for the pet and the owner.

The giving of treats by other persons in the household reflects an expression of affection [14]. In this study, treats are used to reward and influence pet behaviour. Giving human foods to pets during meal times and while food is being prepared is noted in the results, and is presented by owners as being part of the routine of the owner-pet relationship. Owners themselves may receive positive rewards from giving human food to their pet and so the treat-giving is reinforced [17]. This behaviour increases the risk of pet obesity [13, 18, 27]. Owners who use play, rather than food, as a treat are more likely to have normal weight pets [12] and this message needs to be communicated by veterinarians in weight management initiatives.

A lack of perceived control over the pet’s feeding behaviour (such as stealing food) formed a dominant theme in this study. Counselling can be beneficial to help owners build a greater sense of self-efficacy and control in managing their pet’s diet and behaviour. Given the problem of multiple feeders in a household, veterinary practices need to take into account that in a multi-person household, with an overweight pet, more than one person may be responsible for over-feeding the pet. These situations require weight counselling to include all members of the household and not just the actual pet owner. Encouraging a feeding regime that requires only one person to be in control of all the food that the pet receives could be beneficial. Given that owners often fail to complete a weight loss programme [28], lifestyle management, strategies to assist animal owners and continual monitoring of progress are important to maintain owner enthusiasm and cooperation [7, 27].

It is important to recognise owners’ level of perceived control over exercising and the reality of access to open space. In this study, dog owners reported the benefits of allowing their dog to exercise off the lead. However, encountering aggressive or large dogs while out walking is a barrier for exercising a dog, as was negative interactions with people with children [29]. In Ireland, legislation is in place that states that dogs must be kept on a lead at all times in a public place [30]. Encouraging greater pet exercise requires the need for dog owners to have special areas where dogs can be safely allowed to exercise and interact with other dogs without a lead. Recognising a dual obesity epidemic (among owners and their pets), [11] highlight the effectiveness of a combined People and Pets Exercising Together (PPET) programme on both owner and pet weight reduction. There is potential for veterinary services to link with human medical services (including nutritional care services) to adopt a combined approach to pet and owner obesity reduction. Further, there is also potential for veterinary services to advice professionals involved in human health care provision on involving pets in owner weight reduction programmes [11, 31].

### Limitations in the study design and recommendations for future research

Focus groups were deemed the appropriate method for data collection. This type of group discussion enabled pet owners to share and compare experiences and opinions [32, 33]. There were some limitations to the study. The sample was not stratified by gender, and this led to an over representation of female owners. The sample was not stratified by socio-economic group, though different geographical locations, urban and rural, were chosen to minimise this bias. Recruitment was through private veterinary practices, and therefore, it is probable that participants were more engaged in their pet’s health.

Theory of Planned Behaviour (TPB) has been applied to understanding pet owner intentions and their behaviour towards their pets [17, 34]. According to TPB, behavioural change requires changes in attitude and the use of behaviour-change techniques. TPB was not applied to this study; however, the research findings are consistent with the TPB in that behavioural beliefs and control beliefs do appear to underlie some of the participants’ self-reported behaviour.

At the time of research, there was no prevalence rate for pet obesity in Ireland. In the absence of this, the authors recommend that an indicator of prevalence be conducted, so as determine the extent of the problem. Results from this study would add an insightful dimension to a measurement of the prevalence of obesity.

## Conclusion

Pet exercise is influenced by beliefs about pet specific exercise needs, and the implications of exercising one’s pet for the pet owner. Understanding owner behaviour on feeding and exercise allows for a more targeted approach to preventing and treating pet obesity.

## References

[1] Edney A, Smith P (1986). (1986). Study of obesity in dogs visiting veterinary practices in the United Kingdom. Vet Rec.

[2] Lund E, Armstrong P, Kirk C, Klausner J (2005). Prevalence and risk factors for obesity in adult cats from private US veterinary practices. Int J Appl Res Vet M.

[3] Lund E, Armstrong P, Kirk C, Klausner J (2006). Prevalence and risk factors for obesity in adult dogs from private US veterinary practices. Int J Appl res vet M.

[4] McGreevy P, Thomson P, Pride C, Fawcett A, Grassi T, Jones B (2005). Prevalence of obesity in dogs examined by Australian veterinary practices and the risk factors involved. Vet Rec.

[5] Colliard L, Ancel J, Benet J, Paragon B, Blanchard G (2006). Risk factors for obesity in dogs in France. J Nutr.

[6] Colliard L, Paragon B, Lemuet B, Bénet J, Blanchard G (2009). Prevalence and risk factors of obesity in an urban population of healthy cats. J Feline Med Surg.

[7] Sloth C (1992). Practical management of obesity in dogs and cats. J Small Anim Pract.

[8] Klinkenberg H, Sallander M, Hedhammar Å (2006). Feeding, exercise, and weight identified as risk factors in canine diabetes mellitus. J. Nutr.

[9] Zoran D (2010). Obesity in dogs and cats: a metabolic and endocrine disorder. Vet Clin north am small Anim Pract.

[10] Laflamme D (2012). Companion animals symposium: obesity in dogs and cats: what is wrong with being fat?. J Anim Sci.

[11] Kushner R, Blatner D, Jewell D, Rudloff K (2006). The PPET study: people and pets exercising together. Obesity (silver spring).

[12] Kienzle E, Bergler R (2006). Human-animal relationship of owners of normal and overweight cats. J Nutr..

[13] Robertson I (2003). The association of exercise, diet and other factors with owner-perceived obesity in privately owned dogs from metropolitan Perth, WA. Prev Vet Med.

[14] Gibbs O (2008). The growing problem of canine obesity: approaching the owner. Ir Vet J.

[15] Roudebush P, Schoenherr W, Delaney S (2008). An evidence-based review of the use of therapeutic foods, owner education, exercise, and drugs for the management of obese and overweight pets. J Am Vet Med Assoc.

[16] Courcier E, Thomson R, Mellor D, Yam P (2010). An epidemiological study of environmental factors associated with canine obesity. J Small Anim Pract.

[17] Rohlf V, Toukhsati S, Coleman G, Bennett P (2010). Dog obesity: can dog caregivers’ (owners’) feeding and exercise intentions and Behaviours be predicted from attitudes?. J Appl Anim Welf Sci.

[18] Kienzle E, Bergler R, Mandernach A (1998). A comparison of the feeding behaviour and the human-animal relationship in owners of normal and obese dogs. J Nutr.

[19] Bland I, Guthrie-Jones A, Taylor R, Hill J (2010). Dog obesity: veterinary practices’ and owners’ opinions on cause and management. Prev Vet Med..

[20] German A, Morgan L (2008). How often do veterinarians assess the bodyweight and body condition of dogs?. Vet Rec.

[21] White G, Hobson-West P, Cobb K, Craigon J, Hammond R, Millar K (2011). Canine obesity: is there a difference between veterinarian and owner perception?. J Small Anim Pract.

[22] Downes MJ, Devitt C, Downes MT, More SJ (2015). Neutering of cats and dogs in Ireland; pet owner self-reported perceptions of enabling and disabling factors in the decision to neuter. PeerJ.

[23] Attride-Stirling J (2001). Thematic networks: an analytic tool for qualitative research. Qual Res.

[24] White GA, Ward L, Pink C, Craigon J, Millar KM (2016). “Who’s been a good dog?” – owner perceptions and motivations for treat giving. Prev Vet Med..

[25] Wensley S (2008). Animal welfare and the human-animal bond: considerations for veterinary faculty, students, and practitioners. J Vet Med Educ.

[26] Sanderson S, Finco D, Pogrelis A, Stacy L, Unger C (2005). Owner impressions of three premium diets fed to healthy adult dogs. J Am Vet Med Assoc.

[27] German A (2006). The growing problem of obesity in dogs and cats. J Nutr..

[28] Yaissle J, Holloway C, Buffington C (2004). Evaluation of owner education as a component of obesity treatment programs for dogs. J Am Vet Med Assoc.

[29] Cutt H, Giles-Corti B, Knuiman M (2008). Encouraging physical activity through dog walking: why don’t some owners walk with their dog?. Prev Vet Med..

[30] Irish Statute Book. Control of Dogs Regulations S.I. No. 442/1998.1998.

[31] Brown S, Rhodes R (2006). Relationships among dog ownership and leisure-time walking in western Canadian adults. Am J Prev Med.

[32] Kitzinger J (1995). Qualitative research. Introducing focus groups. BMJ.

[33] Gibbs A (1997). Focus groups. Social Research Update.

[34] Toukhsati S, Phillips C, Podberscek A, Coleman G (2012). Semi-ownership and sterilisation of cats and dogs in Thailand. Animals.

